# Edible Insects: Preliminary Study about Perceptions, Attitudes, and Knowledge on a Sample of Portuguese Citizens

**DOI:** 10.3390/foods10040709

**Published:** 2021-03-26

**Authors:** Sofia G. Florença, Paula M. R. Correia, Cristina A. Costa, Raquel P. F. Guiné

**Affiliations:** 1 Faculty of Food and Nutrition Sciences, University of Porto, 4200-465 Porto, Portugal; sofiaguine@gmail.com; 2 Agrarian School of Viseu, Polytechnic Institute of Viseu, 3500-606 Viseu, Portugal; paulacorreia@esav.ipv.pt (P.M.R.C.); amarocosta@esav.ipv.pt (C.A.C.); 3 CERNAS Research Centre, Polytechnic Institute of Viseu, 3504-510 Viseu, Portugal

**Keywords:** edible insects, sustainability, nutritive value, tree classification analysis, knowledge, questionnaire survey

## Abstract

This study investigated the knowledge, attitudes, consumption habits, and degree of acceptability of edible insects (EI) or derived products among Portuguese consumers. This work consisted of a questionnaire survey, undertaken on a sample of 213 participants. For the treatment of data, basic descriptive statistics were used, complemented with chi-square tests to assess some associations between categorical variables. Moreover, a tree classification analysis was carried out using a classification and regression tree (CRT) algorithm with cross-validation. The results indicated that people tend to have correct perceptions about the sustainability issues associated with the use of insects as alternative sources of protein; however, the level of knowledge and overall perception about their nutritive value is low. Regarding the consumption of EI, it was found that only a small part of the participants had already eaten them, doing it mostly abroad, by self-initiative, in a restaurant or at a party or event. Additionally, it was found that the reluctance to consume insects is higher if they are whole, but when they are transformed into ingredients used in food formulations, the level of acceptance increases. Furthermore, men have shown to have a better perception about EI, be more informed about sustainability, and have a higher level of acceptability when compared to women. As a final conclusion, it was observed that the Portuguese still show some resistance to adhere to the use of insects as replacements for meat products, but the market of insect based products can be a good alternative to overpass the neophobia associated with this type of food.

## 1. Introduction

Food is considered a basic necessity for all human beings. However, attending to this need, while sustaining a healthy ecosystem, is still a challenge [1]. Today’s food systems take possession of more than 30% of land, 70% of drinkable water, and 20% of energy, widely contributing to the loss of biodiversity, water, and land at a global scale [1,2]. In order for the food production of present and future generations to be conceivable, it is necessary to make the food systems sustainable, so as to ensure that at economic, social, and environmental levels, it will be possible to generate safe and nutritious foods for all the population [2,3]. Sustainable food systems must ensure food security and nutrition at global scale, while guaranteeing the food security and nutrition for the future generations [3]. It is known that the climate changes deeply impact the primary production, both animal and vegetable farming, while also affecting the marine resources and fisheries. One of the possible approaches to this problem is to focus on the food production by means of new innovative technologies. Additionally, it must also be given high attention to agricultural practices, which still depend strongly on fossil fuels [4].

The expansion of the beef production has led to a considerable environmental impact through the devastation of tropical rain forests, transforming them into pastures for cattle. On the other hand, there is the problem of the greenhouse gas (GHG) emissions. To this matter, there are opposite points of view; while some consider livestock production highly responsible for the increase in GHG [5,6,7], others [8] consider that range livestock production can in fact be more efficient in terms of environment and energy resources, when compared with other food production systems based on land. However, this discussion is not consensual [5,6,7].

The protein intake is essential at the nutritional, economic, and environmental levels. Nevertheless, the livestock sector has a disproportional impact on the loss of biodiversity, drinkable water, and others. Available data show that, at a global level, this sector produces about 15% of the total of the greenhouse gases, being the majority produced by ruminants. The production of 1 kg of beef requires 50 times more land and originates 100 times more GHG than the production of 1 kg of vegetables [2,5,9,10].

The positive impact on the sustainability depends greatly on a significant change on nowadays’ food diets [2]. With the fast growth of the population, and consequent rise of the animal protein consumption, it is paramount to find an alternative that is more sustainable, and therefore avoid such a great degradation of the environment. One of the ideas that has been gaining prominence is the mass production of edible insects (EI). This concept has been highlighted, not only due to the high efficiency that these animals have in converting organic matter in protein, but also for their nutritional value and possible use as animal feed [10,11].

There are about 2100 species of insects that are consumed by humans in Latin America, Asia and Africa. When we compare 1 g of protein, the chicken requires two to three times more land and 50% more water than, for example, mealworms, and when we use beef as a reference, this requires 8 to 14 times more space, and five times more water [10,12]. Regarding greenhouse gases, the chicken releases about 32% to 167% more emissions and the beef 6 to 13 times more CO_2_ equivalents than mealworms [10]. Beyond the fact that EI have a lower environmental impact, the conversion of feed into protein is higher. As an example, the chicken converts about 33% of its diet into edible body mass, whereas mealworms convert about 22% to 45%, the black soldier fly larvae about 43% to 55% and Argentinean cockroaches between 51% and 88% [10,12].

One other aspect of EI is their nutritional richness. Insects are an excellent source of energy, protein, fat, and minerals. However, the nutritional composition depends on the species, the development stage, diet, processing, and other factors. The protein content varies between 40 to 75 g per 100 g, the fat content between 7 and 77 g per 100 g, and the minerals between 3 to 8 g per 100 g. Regarding vitamins, the insects are good sources of biotin, riboflavin, and pantothenic acid, but low in retinol [13,14]. Insects also have high contents of potassium, calcium, iron, magnesium, and selenium [15].

Despite of all the nutritional and sustainable characteristics, there is still some reserve and neophobia when trying to make insects a part of the western diets [13,16]. There are scientific investigations dedicated to the study of the acceptability of European consumers in turning EI a part of the daily diet. These studies concluded that there is a low degree of acceptability, and that those who were more receptive in trying were young males with a high educational degree and with particular interest in food sciences and entomology [16]. One other study showed that there was a higher consumer’s acceptability of food enriched with insect flour than foods that had whole insects [13]. However, none of these studies was carried out in Portugal, and to the best of our knowledge, the study about the habits regarding the consumption of EI, their acceptability or knowledge about their properties and effects has never been conducted in Portugal.

Having into account all the numerous qualities of edible insects, and considering that there is not any published information describing a questionnaire survey about consumption of EI in Portugal in scientific databases, such as ScienceDirect, for example, this study intended to understand what is the level of knowledge, attitudes, and degree of acceptability of the Portuguese population regarding the consumption of EI and its derivatives, in order to build an implementation strategy for possible introduction of this type of product in today’s market and diet. In particular, our research questions were as follows: do Portuguese people eat EI? With what frequency and on which circumstances? Are the Portuguese informed about the sustainability issues regarding their production and consumption? And what is the level of information about the nutritive and/or anti-nutritive qualities of EI? Are the Portuguese prone to the consumption of EI or products containing them? This study was a part of the project “FZ—Drone Flour [FZ—*Farinha de Zângão*, in Portuguese]”, which is being carried out to study the technologic possibility to produce drone flour obtained by the Portuguese beehive holders, as a way to improve their income and also control the Varroa mite in the beehives.

## 2. Materials and Methods

### 2.1. Instrument and Data Collection

The instrument used in the present study was a questionnaire that was purposely developed for this research, and therefore all the questions were formulated based on a discussion methodology, from the research team of the project FZ, previously mentioned. The questionnaire was prepared and then submitted to the approval of the Ethical Commission at the Polytechnic Institute of Viseu, being approved for application under reference 06/SUB/2020.

The questionnaire included different sections as follows: (I) sociodemographic data; (II) characterization of participants’ habits; (III) perceptions about EI and derived products; (IV) knowledge about EI and sustainability; (V) knowledge about nutritive value of EI; (VI) attitudes toward EI and derived products. In Section II, four questions were included that aimed to understand the habits of the participants regarding eating out, in what type or restaurant and with which frequency, as well as the types of foods they prefer when eating out, including ethnic or gourmet food. These aspects were considered to be related with the possible consumption of EI. Section III included eleven questions to evaluate how the participants perceived EI, considering that Portugal is a country without any entomophagy tradition, and addressed aspects such as the adaptability of EI for human consumption, and recognition of their usage, for example. Section IV included seven questions to assess the level of knowledge about aspects related to sustainability, such as, for example, their efficiency in conversion resources into protein or lowest environmental impacts as compared with other animal proteins. Section V included eleven questions to assess the level of knowledge about the nutritional properties of EI, including aspects linked with the macro and micronutrients’ contents, as well as possible anti-nutrients, and their effect on the human health. Finally, Section VI included thirteen questions that related with the consumption of EI and level of acceptance or rejection of EI or EI based products, as well as the motivations for their consumption.

The questionnaire was applied to a convenience sample, chosen according to the facility of recruitment and willingness to participate. Convenience samples, although having some limitations, have also some advantages, like easiness of recruitment, and they can be a good tool for exploratory research [17,18].

The sample size was calculated assuming the following assumptions: a 90% confidence interval, corresponding to a level of significance of 10% and a z score of 1.645, and the power of the test was corresponding to an error of 5%, meaning that the minimum acceptable probability of preventing type II error was 0.05 [19,20]. The Portuguese population in 2019 (the latest year available) was 10,283,822 people, of which around 7.5 million were adults (18 years old or older) [21]. The target population was considered as 25% of the adult Portuguese population. Under these conditions the sample size was calculated as 203 adult individuals [22,23], being this the minimum number of participants for the present research to be statistically meaningful.

The data were collected between September and October 2020, using an internet platform. The questionnaire was disclosed to people by different internet tools, such as e-mail and social networks. Only adult citizens were included in the survey, i.e., aged 18 years or older. Other inclusion criteria were the access to internet and have a computer or other devices available where they could access the questionnaire, as well as the necessary skills to be able to use the computational means used to collect the data. The participation was voluntary and confidentiality of all answers was guaranteed to the participants, who had to provide informed consent to participate in the study. All ethical issues were respected when designing and applying the questionnaire.

### 2.2. Data Analysis

Exploratory analysis of the data was performed using SPSS software V26 (IBM, Inc., Armonk, New York, NY, USA) and Excel 2016. Additionally, the crosstabs tool coupled with the chi-square test were used to evaluate if there were significant relations between some of the categorical variables studied, considering a level of significance of 5%. The coefficient Cramer’s V was used to measure the intensity of the relations found between the studied variables. The value of V varies in the range from 0 (no association) to 1 (perfect association) so that the strength of the association is: V ≈ 0.1 is weak, V ≈ 0.3 is moderate and V ≈ 0.5 or higher is he strong [24].

In the analyses, some items had to be reversed so as to make them in line with the same measurement trend of the rest of items in the same block. Additionally, mean values of item groups were also calculated and variables were recorded in order to assess the targeted types in each case.

The variables measuring the knowledge about EI and sustainability and about the nutritive properties of EI were classified according to the relative influence of the sociodemographic variables, following a tree classification analysis. A Classification and Regression Trees (CRT) algorithm with cross-validation was used, with a minimum change in improvement equal to 0.005 and a minimum number of cases for parent and child nodes of 30 and 10, respectively.

## 3. Results and Discussion

### 3.1. Sample Characterization

The participants that partook in this survey were aged between 18 and 80 years old. The average age of the participants was 43 years old, being the women on average a little younger than men (42 against 47 years old) (Table 1).

In this questionnaire, from a total of 213 respondents, the majority were women (79%) and only 21% were men (Table 2). Regarding the age group, most participants were middle aged adults (31–50 years) (39%), followed by senior adults (51–65 years) (32%), young adults (18–30 years) (24%), and elderly (≥66 years) (4%) (Table 2). Concerning other sociodemographic characteristics, the majority of the respondents were from a high education level (university 40% and post-graduate 38%), living in an urban area (63%), married (60%), and working in a professional area related with nutrition, food, agriculture, environment, biology, or health (60%) (Table 2).

### 3.2. Characterization of Food Habits

The frequency of eating at restaurants was investigated based on the following scale: seldom = less than once/month, sporadically = between once/week and once/month, occasionally = about once/week, moderately = 2 to 3 times/week, frequently = 4 or plus times/week. The results obtained showed that the participants sporadically (36%) and seldom (33%) eat at restaurants, but some do it occasionally (19.7%). People who do it more times are fewer, with only 7.5% of the participants eating at restaurants moderately and 3.7% doing it frequently. When eating at restaurants, most prefer to eat Portuguese traditional food (70%) and ethnical food such as Chinese, Italian, Mexican, Indian, and others (36%), while gourmet meals are preferred by only 11%. Convenience food, including fast food, is the choice for 22%, but 10% do not have a preference and about 6% prefer other types not mentioned explicitly in the questionnaire.

**Figure 1:**
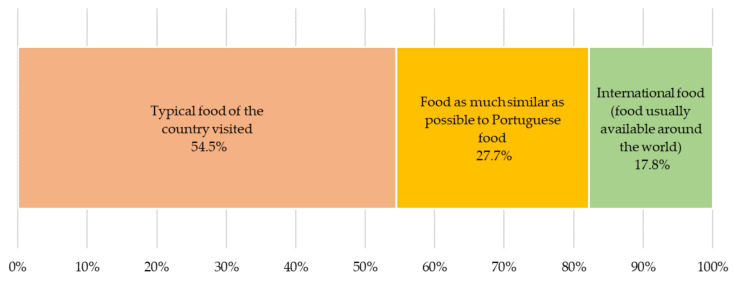
When traveling abroad, what type of food is preferred (legend: rarely = about once/year, occasionally = about 2 to 3 times/year, frequently = plus than 3 times/year).

Of the people who answered the survey, 58% travel abroad rarely (about once/year), 21% occasionally (about 2 to 3 times/year), 8% frequently (plus than 3 times/year), and 13% never travel outside their country. When travelling abroad (Figure 1), the majority prefer to eat food that is typical of the visited country (55%) followed by food that is similar to the Portuguese cuisine (28%) and then international food (18%).

### 3.3. Perceptions about EI and Derived Products

In Table 3 are shown the items questioned and the corresponding opinion of the participants related to statements about EI and derided products. Most participants had no opinion (27.7%) or neither agreed nor disagreed (24.9%) with the statement about the number of edible insects consumed by humans (Item 1). Lucas et al. [15] mention that there are over 2100 species of edible insects consumed all around the world by more than 2 billion people. For Item 2, the majority of the respondents agreed (15.5%) or totally agreed (36.2%) that entomophagy is the act of eating and consuming insects, beginning with the earliest known hominids [12,15].

Insects can be consumed as a whole or as part of a derived product, such as flours or extracts, which most people who answered the survey agreed (20.2%) or totally agreed (33.3%) with Item 4 [14]. The practice of eating insects is very common in many cultures and even seen as a delicacy, with each insect having its own individual flavor, texture, and visual characteristics [13,25]. There are some books that compiled information about different recipes on how to prepare certain insects, since different species have particular gastronomic adaptability [13]. In Item 6 the grand majority of respondents demonstrated not having an opinion (27.2%), or not agreeing nor disagreeing regarding the use of EI by European gourmet restaurants; and concerning insects as being part of the gastronomic culture of countries around the world (Item 7), the preponderance was to disagree (26.8%) or totally disagree (16.0%).

In regards with Item 10, the vast majority of the participants had no opinion (39.4%) when concerning specific legislation for this food group. Regarding EI, these are not explicitly addressed in European legislation, falling under the category of Novel Food (Regulation 2015/2283) [26] which creates room for interpretation of the present legislation, among Member States. Countries, such as Belgium and Netherlands, have created specific regulation for these products, but in Portugal there is no specific legislation in this area [27].

All the items in the group about perceptions (shown in Table 3) were used to calculate a mean value, after reversing items number 5 and 10 in order to have a uniform measurement of the whole group. This variable was then recoded into incorrect perceptions (average score under 3) and correct perception (average score higher than 3). The results for the global sample revealed that 2.3% of the participants did not manifest any opinion about any of the 11 items questioned, 51.7% showed an incorrect perception about EI and derived products and 46.0% showed a correct perception.

In Table 4, it is demonstrated that the only variable that has an association with the perceptions about EI and derived products is the sex of the participants.

### 3.4. Knowledge about EI and Sustainability

The opinions of the participants regarding EI and sustainability are present in Table 5. For the first item, the majority of respondents agreed (23.0%) or totally agreed (39.0%) that insects are a viable protein source. van Huis et al. [10] indicate that with the increase of population worldwide comes a higher demand for protein with an expected increase in the consumption of meat products by more than 75% in 2050. However, the production of livestock is one of the most important contributors to greenhouse gas emissions, with emissions of 9% carbon dioxide, 35–45% methane, and 65% ammonia [12]. Beyond the climate significance, this sector also has a great impact on the environment and biodiversity, being responsible for the loss of usable land, freshwater depletion, and soil degradation [2]. With the increasing population and consequent demands for protein, allied to the impact of the animal production sector, insects have been proposed as a sustainable alternative source of protein. EI, apart from being extremely efficient in converting organic matter into protein, have other advantages over livestock farming, such as having fewer emissions of greenhouse gas, less need of land, water, and lower impact in the biodiversity [10,11,12], which the most participants agreed or totally agreed with, in Items 2 (21.6% and 24.9%) and 3 (21.1% and 28.6%).

Finally, Items 4, 5, and 6 had the highest percentage of no opinion from the participants in the survey (34.7%, 38.0%, and 34.7%, respectively). It has been shown that 1 g of chicken protein requires 2 to 3 times more land and 50% more water than, for example, mealworms, and when we use beef as a reference, this requires 8 to 14 times more space and 5 times more water than mealworms. Regarding greenhouse gases, the chicken releases about 32 to 167% more emissions and the beef 6 to 13 times more CO_2_ equivalents than mealworms [10].

All the items in the group for Knowledge about EI and Sustainability (KEIS) issues (shown in Table 5) were used to calculate a mean score, after reversing item number 5. This variable was named KEIS and was then recoded into low knowledge (average score ∈ [1, 2.5]), moderate knowledge (average score ∈ [2.5, 3.5]) and high knowledge (average score ∈ [3.5, 5]).

Regarding this set of statements, a non-negligible part of participants, 38.0%, did not manifest any opinion about any of the 7 items questioned. Nevertheless, an interesting percentage (30.5%) revealed a high knowledge about the sustainability issues related with EI, while only 8.5% revealed a low knowledge and 23.0% had a moderate knowledge.

**Figure 2:**
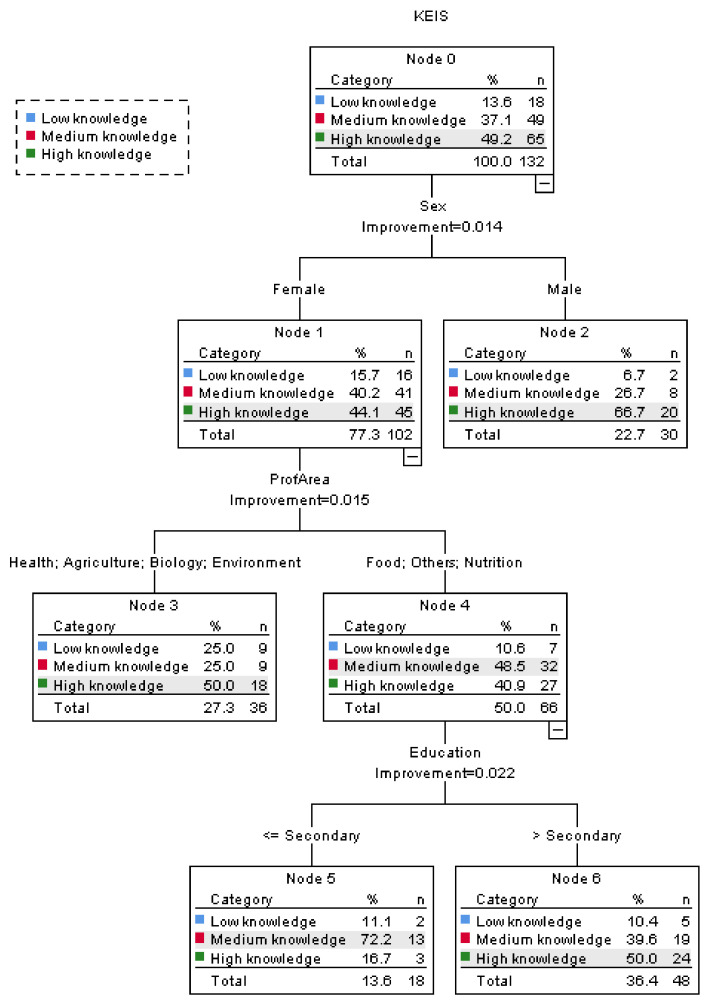
Tree classification for the influence of sociodemographic variables on the level of knowledge about edible insects (EI) and sustainability (KEIS).

The variable KEIS was submitted to a tree classification analysis to evaluate the relative importance of the influential sociodemographic variables: age class, sex, education, living environment, marital status, or professional area. The obtained tree is presented in Figure 2 and contains three levels and seven nodes, of which four are terminal.

The node zero in Figure 2, corresponding to the whole sample, indicates that nearly half of the participants, 49.2%, demonstrated a high knowledge about the sustainability aspects linked with EI, and those who revealed a low knowledge were minority, only 13.6%. These results indicate that, in general, the participants have correct ideas about the topics addressed in the items discussed above. The tree in Figure 2 also reveals that sex was the first discriminating variable, indicating that gender differences are important in terms of the level of knowledge about EI and sustainability. With this regards, men showed to be better informed (66.7% has a high knowledge) as compared with women (only 44.1% had a high knowledge). For the female participants, the next discriminating variable was professional area, thus separating people with professions related with health, environment, biology or agriculture as showing a higher level of knowledge (50.0% of the participants with high knowledge) when compared with other professions, including those related with food or nutrition (40.9% of high knowledge). For this group, the next level was defined by discrimination according to variable education, clearly showing that more educated people, with secondary school or university degree, revealed a higher level of knowledge, thus being more aware of the sustainability aspects linked with EI. This tree revealed that from the tested sociodemographic variables, those influencing more deeply the knowledge about EI and sustainability were in decreasing order sex, professional area and education. In a work that reported a tree classification for the influence of sociodemographic variables on the knowledge about dietary fiber and foods, sex was also identified as the first discriminating variable, followed by education and also living environment [28]. Moreover, in a work by Cardoso et al. [29], variables, such as education and professional area, were also decisive discriminants in a tree classification analysis for the variable in the ambit of their research, which was the perception about healthy eating, followed by age and country. The tree classification technique was also used to evaluate the influence of sociodemographic variables on the consumption patterns of some fiber-rich foods, namely fruits, vegetables and cereals, in different countries, and their results indicated that sex, along with country, were the most relevant discriminating factors observed [30].

### 3.5. Knowledge about Nutritive Properties of EI

Concerning the nutritive aspects of EI, in Table 6 is possible to find the expressed opinion of the respondents towards some statements about this topic. The vast majority of the participants agreed (21.1%) or totally agreed (29.1%) that insects are a good source of energy (Item 1). EI have been shown to have high nutritional value, being a good source of energy, protein, fat, minerals, and vitamins. Some species of EI are able to provide more than 750 Kcal per 100 g, which is higher than some foods such as beef or corn. Apart from being a source of energy, EI contain large amounts of protein, from 40 to 75 g per 100 g, being often, higher than that of soybean and similar to that of chicken and fish. The essential amino acid content can differ between species. While some have all the essential amino acids, others have lysine and tryptophan as the limiting factor [12,13,15]. As for Item 4 the respondents agreed (16.4%) or totally agreed (24.9%) that insects are a good source of protein; however, concerning the quality of the protein (Item 5), most participants had no opinion (36.6%).

Considering Items 3 and 6, concerning, respectively, the vitamins and mineral content of insects, the people who answered the questionnaire had, in their majority, no opinion (48.4% and 36.6%, respectively) or did not agree nor disagree (25.8% and 21.1%, for items 3 and 6, respectively). In this regard, EI have been shown to be rich in both vitamins and minerals, having high amounts of riboflavin, pantothenic acid, biotin, potassium, selenium, magnesium, iron, and calcium [12,13,15].

The fat content varies between 7 and 77 g per 100 g of insect, having a ratio of saturated/non saturated fatty acids below 40%, which, when compared to that of the chicken and fish, is considered better. They are also rich in polyunsaturated fatty acids, like linolenic and linoleic [12,13,15]. On this matter (Item 7), most respondents did not agree nor disagree (20.7%) or had no opinion (39.9%) on the topic.

For Items 8, 10, and 11 concerning, respectively, bioactive compounds, antioxidants, and anti-inflammatory properties of insects, most participants chose not to manifest an opinion on the matter, with percentages ranging from 40.4% on Item 8 to 45.5% on Item 11. In fact, apart from the macro and micronutrient content, EI have been shown to have antioxidant capacity due to certain bioactive compounds present in insect protein hydrolysates and antimicrobial activity as they are a good source of antimicrobial peptides [15].

However, some EI are also a source of anti-nutrients, such as oxalates, hydrogen cyanides, phytic acid and tannins, which can occur naturally in foods but can compromise the digestion, absorption and utilization of certain nutrients [12,14,31]. Regarding this aspect, (Item 9), the bigger percentage of participants declared not having an opinion (52.1%).

Globally, this section of the questionnaire, concerning the nutritional properties of EI, was the part in which most participants showed not having an opinion on the subject.

All the items in the group for Knowledge about Nutritive properties of EI (KNEI) (presented in Table 6) were used to calculate a mean score, after reversing items number 2 and 5. This variable was named KNEI and was then recoded like the previous one into the same categories: low, moderate and high knowledge. For this set of statements, a considerably high percentage of participants, 45.1%, did not express any opinion about none of the 11 items questioned, while 13.6% revealed a low knowledge, 26.3% a moderate knowledge and only 15.0% showed a high knowledge about the nutritive properties of EI.

**Figure 3:**
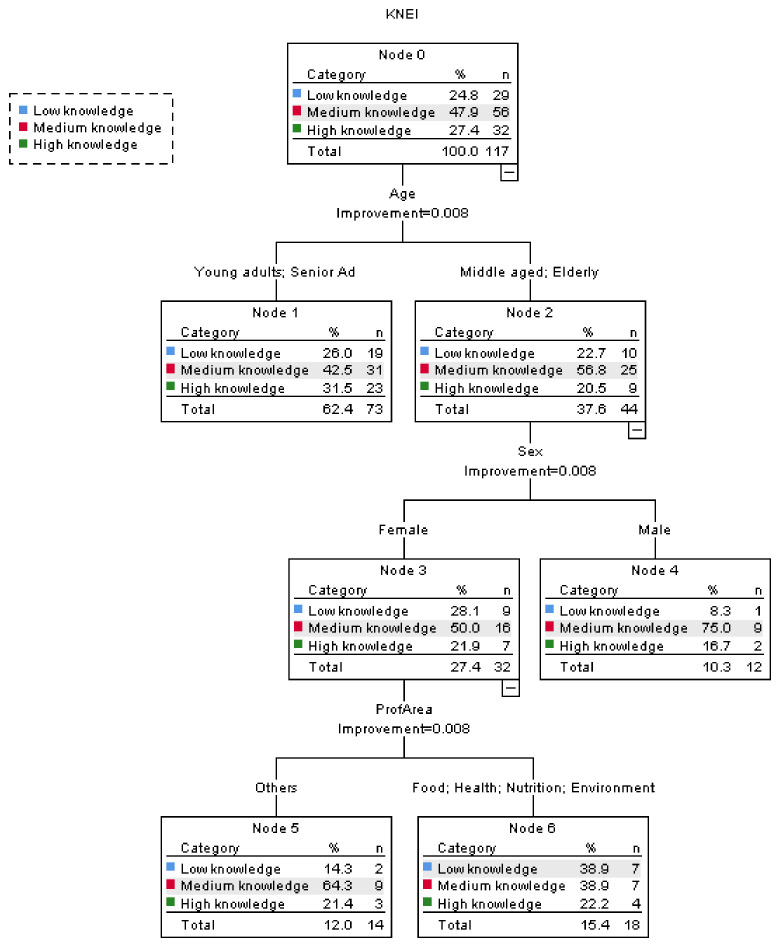
Tree classification for the influence of sociodemographic variables on the level of knowledge about nutritive properties of EI (KNEI).

The variable KNEI was also submitted to a tree classification analysis according to the sociodemographic variables. The obtained tree is presented in Figure 3 and also contains three levels and the same number of nodes as the previous tree for KEIS: seven nodes and four terminal ones. The obtained results showed that the level of knowledge about the nutritive properties of EI is considerably lower when compared with the sustainability issues previously discussed. In fact, for the whole sample at node zero nearly half of the participants, 47.9% had a medium knowledge, and those with a high knowledge were less than one third, 27.4%. For this variable, the first discriminating factors was age and sex was the second for the group of people over 50 years. In this tree level the participants aged under 50 years revealed a higher percentage with a high knowledge, but also a higher percentage with low knowledge, when compared with older people. For the participants aged 51 or more years, sex discriminates between women and men, these last with higher percentage for medium knowledge. For the women, the next discriminating variable was professional area, discriminating people from areas related with food, health, nutrition, and environment from the others. In this last group, 38.9% of the participants revealed a low knowledge, equal percentage was observed for medium knowledge and 22.2% for high knowledge. In the work by Yalçın et al. [28], the tree classification for the influence of sociodemographic variables on the knowledge about dietary fiber and their health benefits, showed that age was also the most important discriminating variable, followed by sex.

### 3.6. Attitudes, Consumption, and Acceptability of EI and Derived Products

We also investigated whether the participants have ever consumed EI, and under which circumstances. The results show that 16.0% of the participants in the survey have already consumed them, while, 67.6% have not and 16.4% do not know or do not remember. Of those who have eaten EI, 61.8% did so abroad and 38.2% in Portugal, having 58,8% of the respondents consumed insects by self-initiative, 29.4% encouraged by friends, 2.9% advised by catering professionals, and 8.8% for other reasons. As for the place where they ate them, 26.5% was in a restaurant, and equal percentage in parties or events. Moreover, consumption occurred at home and in the house of family or friends, for 11.8% and 14.7% of the participants, respectively. Eating them in a hotel has a low expression (only 2.9%), but eating on the street seems also a way to get EI (for 17.6% of the participants).

Regarding the purchase of EI, most participants have not bought the insects themselves (64.7%), or they do not know or do not remember buying them (23.5%), while a smaller percentage (11.8%) actually purchased the EI. From those who bought them, they got them on street markets, 50.0%, in supermarkets, 33.3%, or from others sources (16.7%), but none of the respondents have obtained them on specialized shops or through the internet.

The acceptance of EI can find some resistance in consumers who do not have this food group as part of their culture, creating a certain factor of aversion towards these products [13]. Hence the acceptability of the participants in this survey towards EI as a whole and towards products containing them was also investigated. It was possible to see that the acceptance of whole EI (8.9% would eat and 2.8% would definitely eat), is considerably lower than the acceptance for food products containing EI (23.0% would eat and 12.2% would definitely eat). Participants showed higher rejection for whole EI (35.2% definitely would not eat and 30.5% would not eat), when compared with food products containing EI (19.7% definitely would not eat and 19.2% would not eat)). There was also a high percentage of indifference, for both cases, with a percentage of 25.8% for food products and 22.5% for whole insects. Studies in the area have revealed similar trends, where foods enriched with insects have been more readily accepted by consumers than the insects as a whole [16,32,33].

Regarding the association between several sociodemographic variables and the acceptability of food products containing EI (Table 7), there has been no significant difference between variables except for sex, where men have shown to have a significant higher acceptance than women.

The association between the acceptability of whole EI and sociodemographic variables (Table 8) has shown that only sex and professional area have important associations. Concerning the variable sex, men have shown to have a lower level of rejection than women, whereas, for the variable professional area, people who have jobs related with biology and agriculture demonstrated better acceptance.

**Figure 4:**
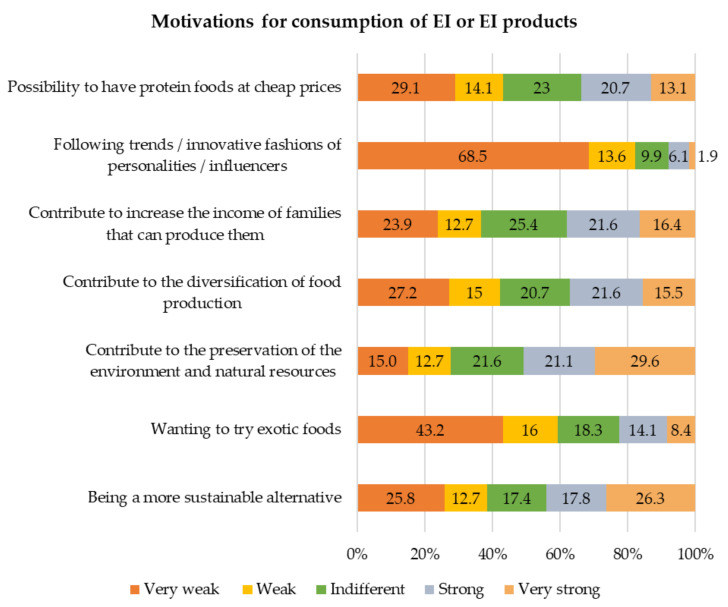
Motivations to encourage the consumption of EI or foods containing EI.

Figure 4 presents the motivations for the consumption of EI by the participants. The main reasons pointed out were the preservation of the environment and natural resources, with 50.7% for strong and very strong motives, being a more sustainable alternative, with 44.1%, and the contribution to the increase in income for the producers’ families, with 38%. The reasons that had less impact on the participants’ encouragement in eating EI were following trends/innovation of personalities/influencers, with 82.4% of weak and very weak reasons, and wanting to try exotic foods, with 59.2%.

## 4. Conclusions

Edible insects have been consumed by people in many countries as an intrinsic part of the gastronomic culture. For some time, EI have been discussed and brought to the surface as a sustainable protein source, since the world population has been, and will continue, to increase at a rate incompatible with the current management of Earth resources. This food group has been shown to have great potential to be a substitute to meat at both nutritional and environmental levels.

The present work has shown some interesting results from a sample of Portuguese citizens, concerning the knowledge, attitudes, and degree of acceptability regarding the consumption of EI. It was demonstrated that about half of the participants had an incorrect perception about EI, with men having a more correct perception than women. Men have also been shown to be better informed about sustainability issues related to insects than women. Regarding the point of view of the nutritional value of EI, a high percentage of participants had no opinion, demonstrating their lesser knowledge as compared with the sustainability of EI.

Additionally, it was found that the majority of the people to whom the questionnaire was applied had never consumed EI, and those who did, did so mostly abroad, by self-initiative and in a restaurant/party/event.

Overall, food products containing EI are better accepted by the participants than the whole counterpart, being men the more receptive to the consumption. The fact that people are less prone to consume whole EI is a common trend also to other foods of animal origin. Finally, it was concluded that the main motives identified by the respondents for the consumption of EI were related to the protection of the environment and sustainability, which are aspects presently highly discussed on the society as well as among the scientific communities and political organizations, at global level.

Based on these findings, the recommendation for possible introduction of EI into the Portuguese food market should start by foods that incorporate EI instead of advancing right away to a possible consumption of the whole counterparts. Additionally, although people in Portugal seem aware of the implications of consuming EI at the sustainability level, concerning their nutritional and health effects, the Portuguese are not yet elucidated, and therefore it is still necessary to increase the dissemination of information about this possible alternative food and its advantages and/or limitations, as compared with other foods, and particular those rich in protein of animal origin.

Some limitations of this study can be highlighted, for example, unequal group distribution, particularly for sex, age, education level or living environment, with the current sample being constituted by less men, less elderly people or with higher education levels. Still, even though having an exploratory character, these findings might be useful for future research, in order to build an implementation strategy of this food group in Portuguese markets, and ideally, in day-to-day diet, thus, helping to overcome neophobia and aversion to EI.

Since this research is still a first approach to the theme of eating EI in Portugal, it would be interesting to proceed with further studies on this area, and in particular to implement a similar study with a more geographical based approach, for example investigating the differences between the interior municipalities in Portugal and the great cities of Lisbon and Porto, or the touristic zone of the Algarve or even the islands of Madeira and Azores, situated on the Atlantic Ocean. Moreover, it would be interesting to apply this or a similar research separately to professionals linked with industry from those in tourism, and see the perspectives from complementary sectors of the society, with different roles and intervention strategies.

## Figures and Tables

**Table 1:** Age of the participants.

Group	Minimum	Maximum	Mean ± SD ^(1)^
Women	18	76	42.11 ± 13.69
Men	19	80	47.47 ± 16.64
Global	18	80	43.24 ± 14.49

^(1)^ Age expressed as mean value ± standard deviation (SD).

**Table 2:** Sociodemographic characterization of the sample at study.

Variable		N	%
Age group	Young adults (18–30 years)	52	24.4
	Middle aged adults (31–50 years)	83	39.0
	Senior adults (51–65 years)	69	32.4
	Elderly (≥66 years)	9	4.2
Sex	Women	168	78.9
	Men	45	21.1
Education level	Basic	0	0
	Secondary	46	21.6
	University	86	40.4
	Post-graduation	81	38.0
Living environment	Urban	138	62.9
	Suburban	28	13.1
	Rural	51	23.9
Marital status	Single	70	32.9
	Married	127	59.6
	Divorced	14	6.6
	Widowed	2	0.9
Professional area	Nutrition	9	4.2
	Food	45	21.1
	Agriculture	22	10.3
	Environment	6	2.8
	Biology	6	2.8
	Health	41	19.2
	None of the above	84	39.4
Total		213	100

**Table 3:** Expressed opinions towards statements related with EI (scale from 1 = totally disagree to 5 = totally agree).

	% of Answers					
Items	1	2	3	4	5	No Opinion
1. There are more than 2000 species of insects consumed by humans in the world.	5.6	8.0	24.9	18.8	15.0	27.7
2. Entomophagy is a dietary practice in which humans consume insects.	402	5.6	16.4	15.5	36.2	22.1
3. Some insects can be used to produce animal feed.	3.3	4.7	13.1	16.9	51.2	10.8
4. There are flours for human consumption produced from insects.	5.2	7.5	16.9	20.2	33.3	16.9
5. In developed countries there is no consumption of insects.	38.0	24.4	13.1	5.6	5.2	13.6
6. Some European gourmet restaurants use edible insects in their culinary preparations.	6.1	13.6	21.6	12.2	19.2	27.2
7. Insects are part of the gastronomic culture of most countries in the world.	16.9	26.8	22.5	8.9	10.8	14.1
8. Insect consumption is characteristic of less developed countries.	16.0	24.9	18.3	14.6	15.5	10.8
9. Not all insects are edible.	4.7	2.3	8.0	12.2	54.0	18.8
10. In Portugal, there are regulations to guarantee food safety in the case of edible insects.	7.5	9.9	13.6	12.6	17.4	39.4
11. Edible insects are used by some people in traditional medicine.	0.5	5.2	12.7	16.9	44.1	20.7

**Table 4:** Associations between the perceptions about EI and derived products and the sociodemographic variables studied.

Variable		N	Incorrect Perception	Correct Perception	CST ^(1)^		CC ^(2)^
					χ^2^	*p*	V
Age group	Young adults	51	54.9	45.1	2.625	0.453	-
	Middle aged adults	81	49.4	50.6			
	Senior adults	67	58.2	41.8			
	Elderly	9	33.3	66.7			
Sex	Women	164	58.5	41.5	9.939	0.002	0.219
	Men	44	31.8	68.2			
Education level	Secondary	43	55.8	44.2	0.234	0.890	-
	University	85	52.9	47.1			
	Post-graduation	80	51.2	48.8			
Living environment	Urban	131	51.9	48.1	1.318	0.517	-
	Suburban	27	63.0	37.0			
	Rural	50	50.0	50.0			
Marital status	Single	69	52.2	47.8	1.961	0.580	-
	Married	123	55.3	44.7			
	Divorced	14	35.7	64.3			
	Widowed	2	50.0	50.0			
Professional area	Nutrition	8	37.5	62.5	7.672	0.263	-
	Food	45	51.1	48.9			
	Agriculture	21	38.1	61.9			
	Environment	6	16.7	83.3			
	Biology	6	66.7	33.3			
	Health	39	59.0	41.0			
	None of the above	83	57.8	42.2			
Pairwise comparisons ^(3)^							
Young adults versus middle-aged adults					*p* = 0.478		
Young adults versus elderly					*p* = 0.042		
Urban versus rural					*p* = 0.124		

^(1)^ CST: Chi square test (level of significance of 5%: *p* < 0.05). ^(2)^ CC: Cramer’s coefficient, only indicated if there were significant differences. ^(3)^ Kruskal–Wallis test (level of significance of 5%: *p* < 0.05).

**Table 5:** Expressed opinions towards statements related with EI and sustainability (Scale from 1 = totally disagree to 5 = totally agree).

	% of Answers					
Items	1	2	3	4	5	No Opinion
1. Insects are a possibility to respond to the growing world demand for protein.	6.6	6.6	18.3	23.0	39.0	6.6
2. The production of insects for human consumption emits about 10 times less greenhouse gases than the production of steak.	3.3	4.2	22.5	21.6	24.9	23.5
3. Insects efficiently convert organic matter into protein.	3.8	3.8	18.8	21.1	28.6	23.9
4. To produce 1 kg of insect protein, 5 times less food is spent than to produce 1 kg of cow protein.	3.3	3.3	19.7	17.4	21.6	34.7
5. To produce 1 kg of chicken protein, 5 times less water is used than to produce 1 kg of insect protein.	13.6	8.5	18.8	12.7	8.5	38.0
6. To produce 1 kg of insect protein requires an area 3 times smaller than to produce 1 kg of pig protein.	4.2	4.2	17.4	16.0	23.5	34.7
7. The ecological footprint of insects is comparatively smaller when compared to other sources of protein for human consumption.	2.3	4.2	15.5	18.8	39.0	20.2

**Table 6:** Expressed opinions towards statements related with nutritive properties of EI (scale from 1 = totally disagree to 5 = totally agree).

Items	% of Answers					
	1	2	3	4	5	No Opinion
1. Edible insects are a good source of energy.	4.2	9.4	20.7	21.1	29.1	15.5
2. Edible insects are poor in macro and micronutrients.	16.0	21.6	18.8	5.6	3.8	34.3
3. Edible insects contain group B vitamins.	2.3	6.6	25.8	6.1	10.8	48.4
4. Edible insects are very rich in animal protein.	2.3	8.0	22.1	16.4	24.9	26.3
5. Insect proteins are of poorer quality compared to those of other animal species.	17.4	18.3	19.7	4.2	3.8	36.6
6. Edible insects contain minerals of nutritional interest, such as calcium, iron and magnesium.	1.4	7.0	21.1	16.0	17.8	36.6
7. Edible insects contain fat, including polyunsaturated fatty acids.	5.6	13.6	20.7	8.5	11.7	39.9
8. Edible insects contain bioactive compounds beneficial to human health.	1.9	8.5	20.2	15.0	14.1	40.4
9. Edible insects contain anti-nutrients, such as oxalates and phytic acid.	6.1	11.3	18.8	6.1	5.6	52.1
10. Some edible insects have a proven antioxidant effect.	2.3	7.5	22.5	8.9	13.1	45.5
11. Some edible insects may have anti-inflammatory activity.	2.8	7.0	20.2	11.3	15.5	43.2

**Table 7:** Associations between the acceptability of food products containing EI and the sociodemographic variables studied.

Variable		N	Rejection	Acceptance	CST ^(1)^		CC ^(2)^
					χ^2^	*p*	V
Age group	Young adults	37	62.2	37.8	2.343	0.504	-
	Middle aged adults	60	51.7	48.3			
	Senior adults	56	46.4	53.6			
	Elderly	5	60.0	40.0			
Sex	Women	123	56.9	43.1	4.270	0.039	0.164
	Men	35	37.1	62.9			
Education level	Secondary	30	60.0	40.0	2.807	0.246	-
	University	69	56.5	43.5			
	Post-graduation	59	44.1	55.9			
Living environment	Urban	101	48.5	51.5	1.879	0.391	-
	Suburban	21	61.9	38.1			
	Rural	36	58.3	41.7			
Marital status	Single	48	60.4	39.6	3.789	0.285	-
	Married	97	49.5	50.5			
	Divorced	11	54.5	45.5			
	Widowed	2	0.0	100.0			
Professional area	Nutrition	8	62.5	37.5	8.075	0.233	-
	Food	30	46.7	53.3			
	Agriculture	19	31.6	68.4			
	Environment	5	60.0	40.0			
	Biology	5	20.0	80.0			
	Health	31	61.3	38.7			
	None of the above	60	58.3	41.7			

^(1)^ CST: Chi square test (level of significance of 5%: *p* < 0.05). ^(2)^ CC: Cramer’s coefficient, only indicated if there were significant differences.

**Table 8:** Associations between the acceptability of whole EI and the sociodemographic variables studied.

Variable		N	Rejection	Acceptance	CST ^(1)^		CC ^(2)^
					χ^2^	*p*	V
Age group	Young adults	40	92.5	7.5	2.780	0.427	-
	Middle aged adults	69	84.1	15.09			
	Senior adults	50	80.0	20.0			
	Elderly	6	83.3	16.7			
Sex	Women	137	87.6	12.4	4.724	0.030	0.169
	Men	28	71.4	28.6			
Education level	Secondary	34	94.1	5.9	4.677	0.096	-
	University	67	86.6	13.4			
	Post-graduation	64	78.1	21.9			
Living environment	Urban	97	84.5	15.5	0.069	0.966	-
	Suburban	25	84.0	16.0			
	Rural	43	86.0	14.0			
Marital status	Single	55	90.9	9.1	4.017	0.260	-
	Married	95	82.1	17.9			
	Divorced	13	84.6	15.4			
	Widowed	2	50.0	50.0			
Professional area	Nutrition	8	75.0	25.0	14.797	0.022	0.299
	Food	38	86.8	13.2			
	Agriculture	16	62.5	37.5			
	Environment	4	75.0	25.0			
	Biology	4	50.0	50.0			
	Health	31	96.8	3.2			
	None of the above	64	87.5	12.5			

^(1)^ CST: Chi square test (level of significance of 5%: *p* < 0.05). ^(2)^ CC: Cramer’s coefficient, only indicated if there were significant differences.

## Data Availability

Data are available from the corresponding author upon reasonable request.
